# Unravelling geospatial distribution and genetic diversity of greenhouse whitefly, *Trialeurodes vaporariorum* (Westwood) from Himalayan Region

**DOI:** 10.1038/s41598-023-37781-y

**Published:** 2023-07-24

**Authors:** Amit Umesh Paschapur, Ashish Kumar Singh, Ramesh Buski, P. N. Guru, B. Jeevan, K. K. Mishra, Lakshmi Kant

**Affiliations:** 1 Division of Crop Protection, ICAR-Vivekananda Parvatiya Krishi Anusandhan Sansthan, Almora, Uttarakhand 263601 India; 2 Division of Entomology, ICAR-Indian Agricultural Research Institute, New Delhi, 110012 India; 3 Division of Entomology, ICAR-Central Institute of Post-Harvest Engineering and Technology, Ludhiana, Punjab 141001 India; 4 Director, ICAR-Vivekananda Parvatiya Krishi Anusandhan Sansthan, Almora, Uttarakhand 263601 India

**Keywords:** Biotechnology, Molecular biology, Plant sciences, Zoology

## Abstract

The Greenhouse whitefly (GWF), *Trialeurodes vaporariorum* (Westwood) (Hemiptera: Aleyrodidae), is a destructive pest that affects protected cultivation worldwide. The Indian Himalayan region is particularly vulnerable to GWF introduction, invasion, and spread due to the expansion of protected cultivation and climate change. In this study, we collected 32 naturally occurring GWF populations, mainly from the Uttarakhand state in the Indian Himalayan region, to investigate the distribution pattern and genetic diversity of *T. vaporariorum*. Our sampling was representative of the region's vegetation diversity and geographical location, and we collected samples from multiple sites within each locality to account for local variations. The mtCOI gene was used to accurately detect and identify GWF and to sequence haplotypes prevalent in the Uttarakhand state. The maximum likelihood method used for phylogenetic studies revealed that all 32 whitefly samples in this study belonged to *T. vaporariorum* and were prevalent in all the collected localities. Our population genetic study using mtCOI showed variation within *T. vaporariorum* populations, with 20 distinct haplotypes present. Notably, haplotype 2 (H2) was the most dominant haplotype among the sampled populations. These results provide fundamental knowledge for understanding the geographical distribution and ecology of *T. vaporariorum* in the Uttarakhand state of the Indian Himalayan region. The discovery of geospatial and genetic diversity of GWF in the Himalayan region underscores the importance of pest alertness, research prioritization, and the development of sustainable management strategies to protect crops.

## Introduction

The Indian Himalaya is a biodiverse region, hosting 30,377 species of protozoa and animalia, representing about 30.16% of the total Indian fauna, with arthropods contributing 26,393 species^1^. Unfortunately, the Indian Himalaya is vulnerable to climate change, which poses a risk of species emergence, outbreak, and extinction, and threatens food security in the region^2^. Protected agriculture, which creates favorable microclimates to protect crops from biotic and abiotic stresses, has emerged as a new paradigm to improve food security, income, and crop protection in the region^3^. Polyhouse structures have been used extensively for commercial cultivation of high-value and off-season vegetables in the Himalayas, but the microclimate under these structures can favor the growth and development of pests, diseases, and soil-borne nematodes, which can threaten crop productivity^4–6^.

Greenhouse whitefly (GWF), *Trialeurodes vaporariorum* (Westwood) (Hemiptera: Aleyrodidae) is a destructive pest problem for protected cultivation globally, infesting over 800 species of plants^7,8^. The wide host range comprises tomato, egg-plant, pepper, cucumber, chrysanthemum and other agriculturally important crops.

Due to its polyphagous nature and potential to act as a virus vector, GWF has become a major scourge for protected cultivation practicing farmers. Adult and nymph of the GWF reduce plant vigor by sucking the sap from foliage, flower, seeds, secreting honey dew, which attracts the development of soot molds fungi^7^. These molds affect photosynthesis in leaves, leading to significant economic loss to the crops^9^.

From India, GWF was first recorded from Nilgiri hills of Tamilnadu, southern state of India^10^ and successively has been reported in other parts of the country^11–14^. The increase in area under protected cultivation and climate change in the Uttarakhand states of Indian Himalayan region are critical factors in vulnerability to introduction, invasion, and emergence of GWF in new areas. Therefore, updating the status of GWF, mapping the geospatial distribution pattern, and intraspecific genetic diversity of the burgeoning population is obligatory to ensure timely mitigation of insect pests through suitable pest management operations. Additionally, keeping an eye on GWF springing into new regions is key in sending alertness to the farming community, safeguarding crops, and ensuring maximum returns to farmers. However, the geospatial distribution and genetic diversity for GWF is unknown from the Uttarakhand states of Indian Himalayan region. Moreover, identification of genetic variation in populations of GWF is a key objective to formulate any management plan and pest management decision making.

Traditional identification of GWF is based on morphological traits, which has several drawbacks, including reliance on expert taxonomists, time-consuming, low resolution in presence of conspecific character, presence of cryptic population, and ergonomic issues^15–17^. DNA barcoding-based species identification utilizing mitochondrial cytochrome C oxidase subunit I (mtCOI) offers rapid, reliable detection and study of genetic variation among GWF^18^. Therefore, in this study, we aimed to use mtCOI gene markers to unravel the distribution pattern and genetic diversity among GWF (*T. vaporariorum*) for monitoring, tackling the pestilence to protect the protected cultivations.

Despite a few genetic diversity and distribution studies on GWF from India^19,20^, no populations and genetic diversity study has been conducted for Uttarakhand states, where protected cultivation is an important agricultural enterprise on a large scale. Therefore, we conducted extensive geographical sampling of GWF from protected cultivations, collecting 32 naturally occurring GWF populations representing Uttarakhand states of India. We aimed to identify the mtCOI gene sequence variation-based haplotypes most commonly representing in the sampled geographical region to formulate any management plan and pest management decision making.

## Results

A total of 32 *T. vaporariorum* whitefly samples were collected from commercial host crops including tomato, capsicum, french bean, brinjal, cauliflower, snake guard, okra, bottle guard, garden pea and ornamentals like salvia, marigold. These crops provided a significant food source and habitat for the greenhouse whitefly (GWF) population in the study area. These crops were widely grown in open and protected structures and were heavily infested by GWF. While the chosen host crops may not be exhaustive in terms of their contribution to the genetic variation of GWF, they were important sources for the pest species. The extensive geographical sampling of GWF populations from protected cultivations conducted in this study aimed to capture a wide range of genetic diversity and distribution patterns among GWF in the Indian Himalayan region. The combination of the chosen host crops and the extensive geographical sampling approach was expected to provide a comprehensive understanding of the genetic diversity and distribution patterns of GWF in the study area.

Most of the samples were collected from Uttarakhand and three samples from Himachal Pradesh, one each from Delhi and Tripura. The partial *mtCOI* gene were successfully amplified and sequenced for molecular identification and phylogeny estimation. The identity of *T. vaporariorum* in collected samples was supported by morphological and molecular diagnostic technique. The result suggests the frequent occurrence of *T. vaporariorum* in the surveyed locality including open and protected structures on range of host crops. The surveys were conducted for the first time in few districts like Chamoli, Dehradun, Nainital and Pithoragarh of Uttarakhand state and greenhouse whitefly infestation was recorded for the first time in commercial crops like Tomato, Capsicum, French bean, Brinjal and Cauliflower.

**Figure 1:**
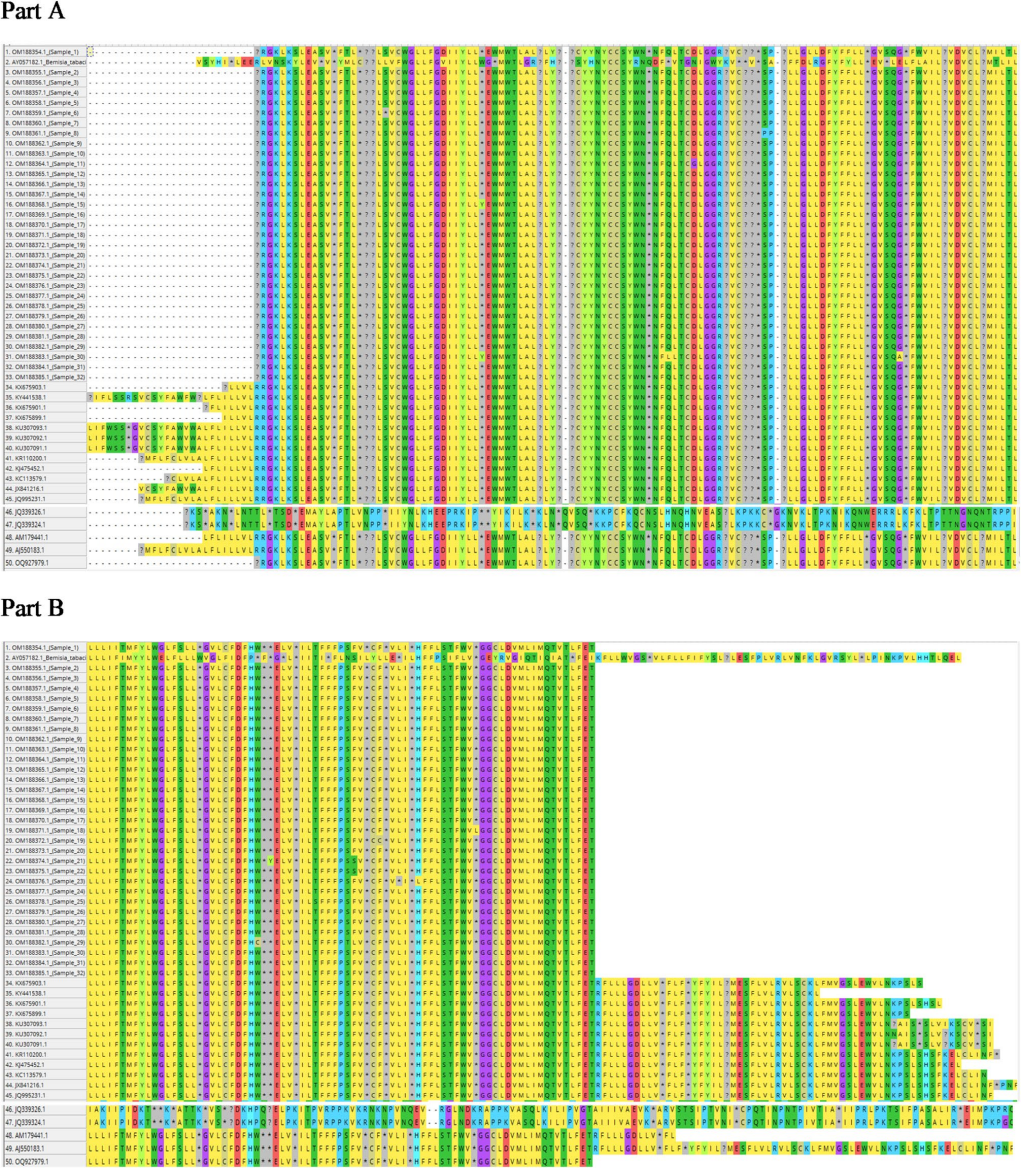
Multiple alignment of COI sequences from *T. vaporariorum*. The figure displays a multiple sequence alignment of COI sequences obtained from *T. vaporariorum*, a species of whitefly. The alignment includes a representative set of sequences derived from different individuals or populations of T. vaporariorum. The alignment highlights the conserved regions and sequence variations among the COI sequences. Conserved residues are indicated by asterisks (*), while amino acid substitutions are represented by the corresponding single-letter code. Gaps (–) are inserted to maintain alignment integrity. The alignment presented in this figure was generated using the MEGA software and represents in two part of the total dataset due to limitation of size of display.

**Figure 2:**
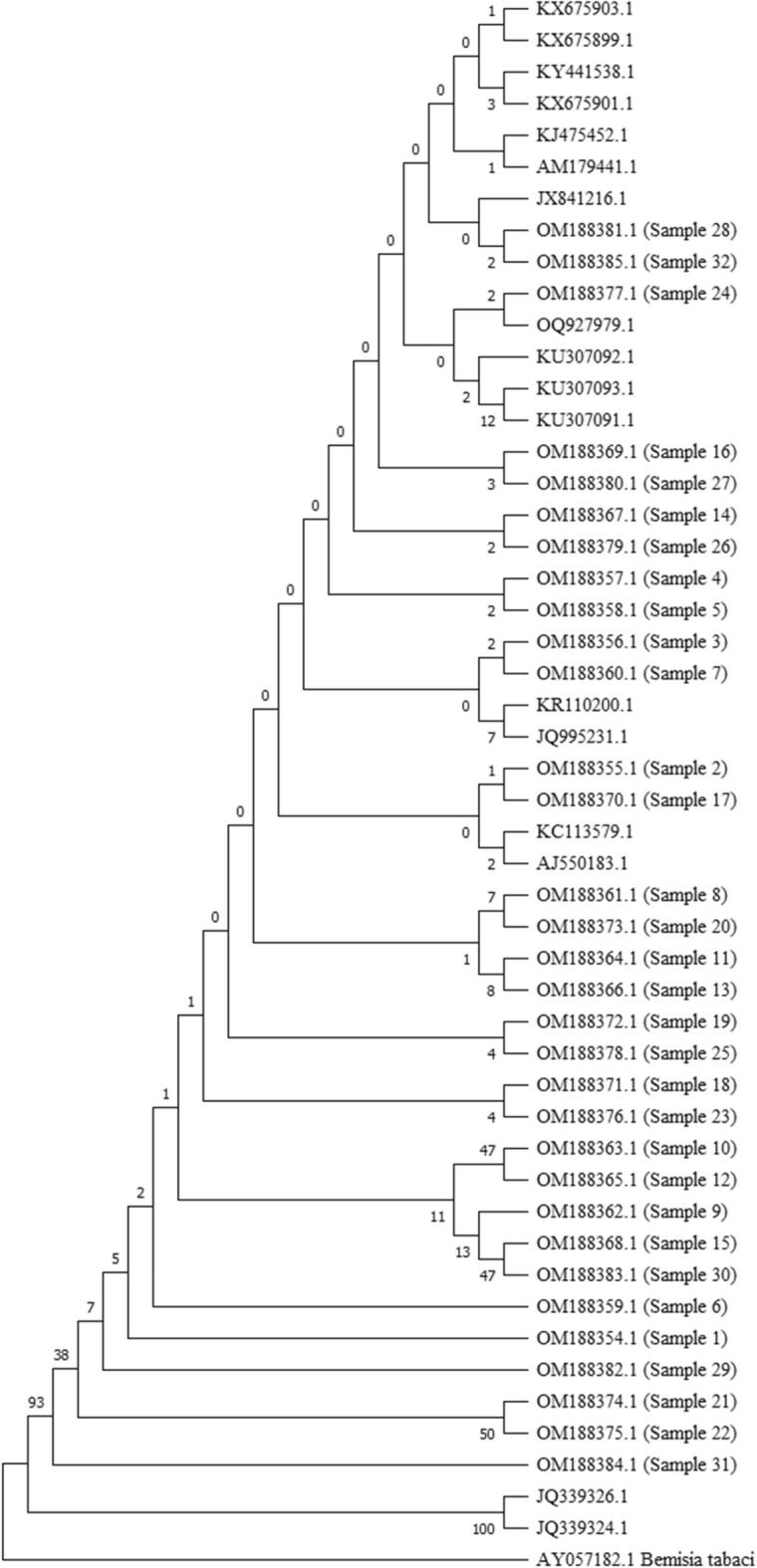
Phylogenetic tree based on mtCOI sequences from *T. vaporariorum*. The figure presents a phylogenetic tree constructed using the maximum likelihood approach with 500 bootstrap replications. The tree represents the evolutionary relationships among *T. vaporariorum* individuals based on their *mtCOI* sequences. In the construction of the tree, a total of 32 partial COI sequences obtained from the present study were combined with reference sequences from public databases and outgroup sequences (AY057182.1) retrieved from GenBank. Each sequence is labeled with a sample name corresponding to the samples collected and studied in this research.

The genetic diversity based on *mtCOI* gene sequences of collected individuals have been submitted in GenBank database citing the information of locality, host range, geospatial data etc. and the accession numbers have been received and summarized in Table 1. The phylogenetic analysis of 32 samples from various agroclimatic regions of the present study were combined by retrieving some available *T. vaporariorum* accessions viz., AY521265, NC006280, LN614547 to exploit as ingroups whereas the Genbank accessions viz., KU761949 *Aleurocanthus camelliae*, NC029155 *Aleurocanthus spiniferus*, AY572538 *Aleurochiton aceris*, NC005939 *Aleurodicus dugesii*, MT880225 *Aleyrodes shizuokensis*, KR819174 *Bemisia afer*, MH205754 *Bemisia tabaci*, AY572539 *Neomaskellia andropogonis*, NC006292 *Tetraleurodes acacia* were used as outgroups. Phylogenetic studies based on maximum likelihood method reveals that all the 32 whitefly samples from the study belonged to *T. vaporariorum* and it is found prevalent in all the collected localities (Figs. 1, 2).

**Table 1:** Distribution and frequency of different mitochondrial haplotypes of *T. vaporariorum* belongs to India.

Haplotype name	Accession number	Number of sequences
H1	OM188354_Hawalbagh_UK	1
H2	OM188355_Almora_UK OM188356_Kafligair_UK OM188357_Doba_UK OM188358_Niglat_UK OM188360_Dwarahat_UK OM188367_Valley_of_flowers_UK OM188377_Pangoot_UK OM188379_Daulaghat_UK OM188380_Lakhwada_UK OM188381_Dhanpau_UK OM188385_Mukteshwar_UK OM188369_Kufri_HP OM188370_Kasol_HP	13
H3	OM188359_Darim_UK	1
H4	OM188361_Govindpur_UK	1
H5	OM188362_Rudradhara_UK	1
H6	OM188363_Pantnagar_UK	1
H7	OM188364_Mussoorie_UK	1
H8	OM188365_Chopta_UK	1
H9	OM188366_Gopeshwar_UK	1
H10	OM188373_Jal_Dhalar_UK	1
H11	OM188374_Mehragaon_UK	1
H12	OM188375_Ranibagh_UK	1
H13	OM188376_Nainital_UK	1
H14	OM188378_Jageshwar_UK	1
H15	OM188382_Vikas_nagar_UK	1
H16	OM188383_Rampur_UK	1
H17	OM188384_Gwaldam_UK	1
H18	OM188368_Kasauli_HP	1
H19	OM188371_New_Delhi_DL	1
H20	OM188372_Lembucherra_TR	1

The variations within the haplotypes of *T. vaporariorum mtCOI* partial gene was observed for groups made out by partitioning samples based on collected regions as Uttarakhand, Himachal Pradesh and Others (New Delhi and Tripura). The number of haplotypes, total number of variable sites, and haplotype diversity, and nucleotide diversity, average number of nucleotide differences, G+C content and total number of mutations were represented in Table 2. A total of 20 haplotypes were identified from the 32 sequences of our study. Haplotype 2 (H2) was found dominant by sharing 13 sequences where in which 11 of them belong to Uttarakhand and 2 of them belong to Himachal Pradesh (Table 1).

**Table 2:** Genetic diversity analysis in haplotypes of *T. vaporariorum* India.

Location	Sequences	Haplotypes	Number of variable sites, S	Haplotype diversity	Nucleotide diversity Pi	Average number of nucleotide differences, k	G+C	Total number of mutations, Eta:
Uttarakhand	27	17	24	0.843	0.00346	1.98291	0.372	24
Himachal Pradesh	3	3	2	0.667	0.00233	1.33333	0.372	2
Others	2	2	2	1.000	0.00349	2.00000	0.373	2

**Figure 3:**
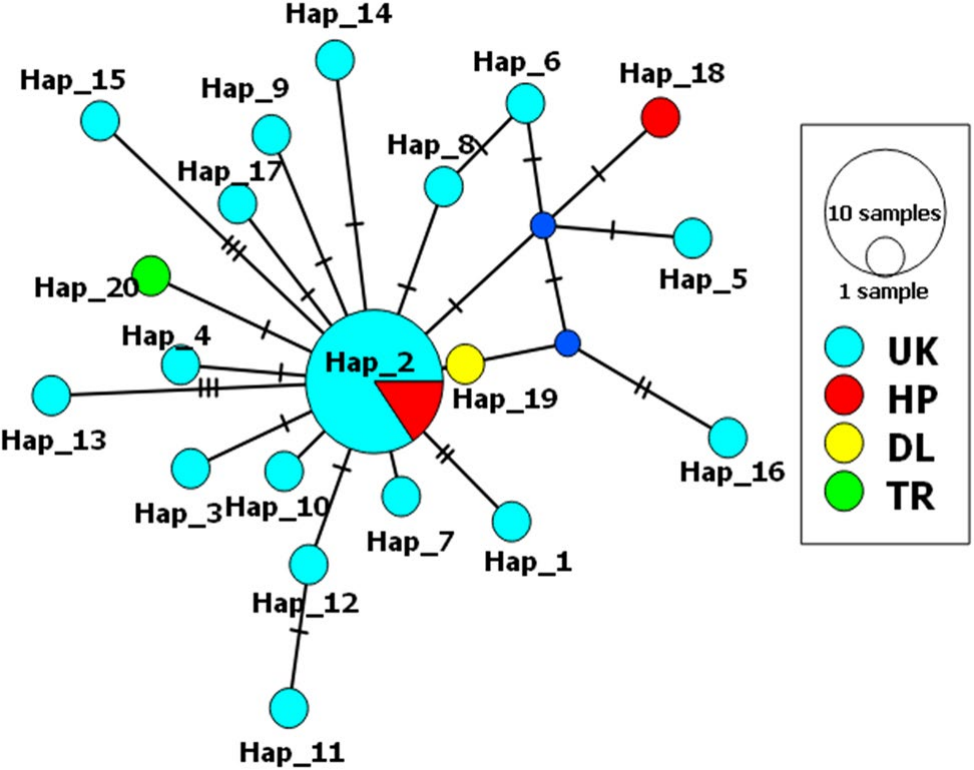
Minimum spanning network for collected samples of *T. vaporariorum* using *PopART* (Population analysis with reticulate trees) software.

Nucleotide diversity of the Uttarakhand observed to be 0.00346 whereas in Himachal Pradesh it was 0.00233 and the group-others (one sequence each of New Delhi and Tripura) was 0.00349. In the same way, haplotype diversity observed higher in others (1.000) followed by Uttarakhand (0.843) and Himachal Pradesh (0.667) (Table 1). The distribution and frequency of different mitochondrial haplotypes of *T. vaporariorum* belongs to India were represented in Table 1. The relationship among the haplotypes of each mitotype was determined by minimum spanning network analysis (Fig. 3). The color code for the groups was represented differently i.e., Red indicates Himachal Pradesh, Violet indicates Uttarakhand, Yellow indicates New Delhi whereas Green indicates Tripura sequence. The 32 samples were diversified into a total of 20 haplotypes that were networked distantly from each other. Haplotype 2 was dominant by sharing 13 sequences and placed in the center of the network. The New Delhi and Tripura sequences form two unique haplotypes i.e., H19 and H20 respectively. Haplotype 2 shares 11 sequences of Uttarakhand and 2 sequences of Himachal Pradesh whereas the remaining haplotypes are unique from Uttarakhand region. The results indicate the variation among collected individuals of greenhouse whiteflies.

## Discussion

The polyphagous whitefly species, *Trialeurodes vaporariorum* Westwood (Hemiptera: Aleyrodidae), is a highly destructive pest of cold climate greenhouses^21^. It has been reported to infest over 102 herbaceous plants belonging to 36 families across the world^22^. The infestation of greenhouse whitefly in the Indian Himalayas was first reported in the 1990s; however, its impact on commercial cultivation of vegetable crops and ornamental plants under protected and open field conditions was only reported after 2000s^23^. Currently, *T. vaporariorum* is classified as one of the key pests of tomato, cucumber, capsicum, french bean, gerbera, salvia and gladiolus under protected environment in the Indian Himalayan region^6^. During our surveys, we observed mixed populations of *T. vaporariorum* and *B. tabaci* in open field conditions; however, *T. vaporariorum* was the predominant species under protected cultivation systems in the Alpine and Humid subtropical climatic zones of the northwestern Indian Himalayas in the states of Uttarakhand and Himachal Pradesh. We also found that *T. vaporariorum* infestation was consistently recorded on all ornamental plants in high elevation locations, such as the Valley of Flowers (Chamoli), with an average pest infestation rate of 15–30%. Given the potential economic impact of *T. vaporariorum* infestations on various crops, it is crucial to develop effective management strategies for this pest. Integrated pest management approaches that combine cultural, physical, biological, and chemical control methods have been found to be effective in controlling *T. vaporariorum* infestations^24^. In addition, the development of resistant plant varieties could also serve as an effective long-term management strategy^25^. It is also important to note that *T. vaporariorum* infestations have been reported in other regions with similar climatic conditions, such as parts of Europe and the United States^26,27^. Overall, our findings highlight the need for continued monitoring and research on *T. vaporariorum* infestations in the Uttarakhand state of Indian Himalayas and other regions with similar climatic conditions.

In the present study, the Uttarakhand state located in Indian Himalayan region of India, was surveyed to detect diversity of *T. vaporariorum* populations. Whitefly survey using aspirator tools are widely used tools to collect whitefly for phylogeography study^28^. The molecular diagnostic technique deployed was convenient to identify whitefly populations and expanding our knowledge of *T. vaporariorum* occurrence and diversity in Uttarakhand state.

Molecular diversity techniques have been proven useful in unravelling diverse insect pest of agricultural importance including new species^29^, biotypes^30^, cryptic species^31^ and haplotypes^32^. The presence of *T. vaporariorum* was recorded most frequently from all the surveyed plant host and respective locations. We have detected *T. vaporariorum* from all the regions ranging from the 300 AMSL (Pantnagar) to the highest altitude 2400 AMSL (Chamoli) of survey. In our study, populations of *T. vaporariorum* were recorded for the first time from higher altitudes having temperate climate including Almora, Nainital, Bageshwar and Chamoli districts. The finding of *T. vaporariorum* in all the thirty-two locations, shows the frequent occurrence (100%) and prevalent of GWF in Uttarakhand state compare to the survey in Karnataka, Tamil Nadu and Andhra Pradesh states of India^19^, Costa Rica^33^. This study suggests that *T. vaporariorum* have successfully colonized and spread in the surveyed region of Uttarakhand irrespective of sampled crop in polyhouses as well open fields. In the Indian Himalayan region, incidence of *T. vaporariorum* have been recorded long back from protected crops of Himachal Pradesh^23^. The presence of *T. vaporariorum* may have harmful impact on yield of protected crops grown in the regions due to agility in adaptiveness, colonization in newer habitats and development of insecticidal resistance^34,35^.

The *mtCOI* gene sequence amplification found useful for the detection of whitefly species. *mtCOI* gene have been used as potential marker in the past for discrimination of white fly species complex, evolutionary and genetic diversity study with accuracy and reliability^36–38^. The phylogenetic tree reconstructed by retrieving and aligning *mtCOI* sequences with the expansion of *mtCOI* supports the relatedness and validity of the molecular identification of collected *T. vaporariorum*.

Population genetic study using *mtCOI* suggest the high variation with presence of 20 haplotype in the *T. vaporariorum* populations. Most of the haplotype differs with single nucleotide mutations with their corresponding haplotypes except the haplotype number 1, 13, 15, 16. Haplotype 2 represents the primitive population and most frequent among all haplotypes. This suggests that the most of the populations are descendants of Haplotype 2 populations. Most of the populations are recently introduced in the area and their dispersal agents haven’t had time to disperse. Since these populations are recently introduced, the decision on regular monitoring of these areas may be recommended before the population reaches economic injury level. Presence of twenty haplotype suggests the high diversification among all the haplotypes. However, *mtCOI* based phylogeny showed the close relation. The obtained results are in consensus with the results of Wainaina et al. and Barboza et al. who reported sampled sequences grouped in monophyletic clade^33,39^. Similarly, Despoina et al., 2015 have shown limited sequence divergence among *T. vaporariorum* populations^37^. Hence, low level of genetic variation may exist in *mtCOI* sequences around the world for *T. vaporariorum*.

The result of this study gives fundamental knowledge on understanding of geographical distribution and ecology of *T. vaporariorum* in Uttarakhand. The host factor and ecological adaptability may be the reason for high abundance in different area^33^. However, the effect of several other factors such as altitudinal variation, temperature effect, crop cycle, rainfall pattern, wind directions, wind speed, dispersal through planting material and existence of natural population could be explored for future studies to get real scenario and cause of distribution. We have revealed here, the wide distribution pattern of *T. vaporariorum* on different host at different altitudes exhibiting uniformity in genetic level. The detection of the whitefly population in Uttarakhand state areas may help us to extend surveillance, monitoring and decision making about *T. vaporariorum* management initiative in the identified areas. Additionally, the present study attracts special attention to study the associated viruses being vectored by *T. vaporariorum* on different host plants.

## Conclusion

Global distribution, polyphagous nature and virus vector potential of Greenhouse whitefly (GWF) *Trialeurodes vaporariorum* (Westwood) is a serious concern for farming community. The spread of GWF into new area may cause mayhem to the crops. Therefore, regular survey, identification, documentation on geospatial distribution of GWF is urgent need. Present study, suggest the frequent occurrence of GWF from Indian Himalayan states. Population genetic study using *mtCOI* suggest the high variation with presence of 20 haplotype in the *T. vaporariorum* populations, where haplotype 2 (H2) was found most dominant. The obtained result draws attention of researchers, policy makers and farmers for pest alertness, decision making research prioritization, development and adoption of sustainable management strategies of GWF to safeguard the crops.

## Materials and methods

### Survey sites and sample collection

**Figure 4:**
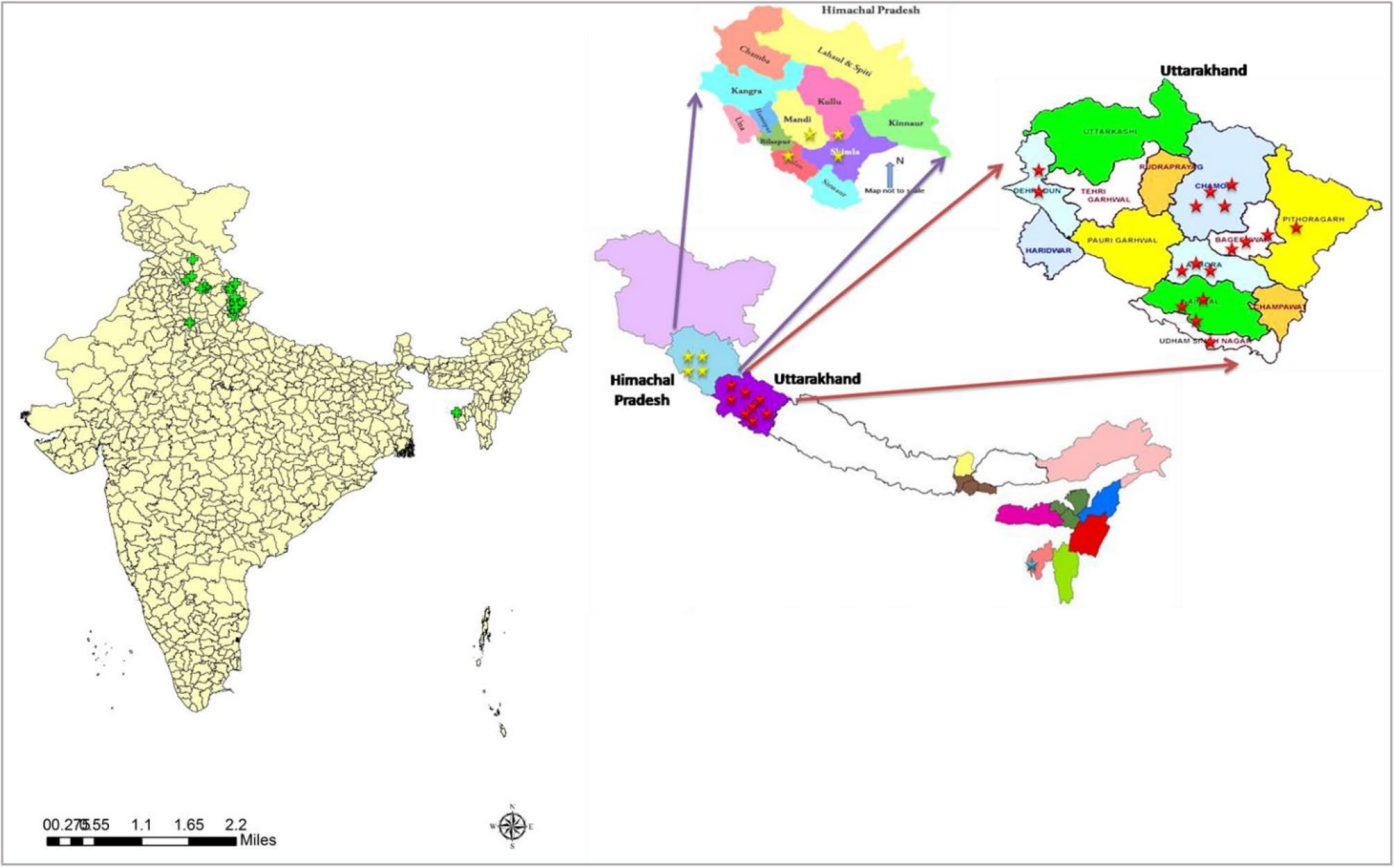
The geographic locations of sample collection sites and distribution pattern of Greenhouse Whitefly, *Trialeurodes vaporariorum* (Westwood) from Indian Himalayan Region. The given map used for indication of sample collection sites and generated using ArcGIS software (version 10.7).

A total of 32 greenhouse whitefly samples were collected from Uttarakhand, Himachal Pradesh, New Delhi and Tripura states during 2019–2021 from both protected cultivation systems as well open field conditions (Table 3). Among which, 27 samples were collected from Uttarakhand state located in Northern India, bordering the Tibet Autonomous Region of China in the north, Nepal in the east, whereas, 3 samples were collected from Himachal Pradesh (Fig. 4). Both These states are part of North western Indian Himalayan region. The remaining two reference samples, one each were collected from New Delhi and Tripura respectively . The field surveys were conducted throughout the year from 2019 to 2021. The selected survey sites in the two states of Indian Himalayan region were at the elevation of 300–2400 m above mean sea level with annual mean rainfall ranging between 750 and 1600 mm and mean minimum and maximum temperature ranging between 4.6–8.9 °C and 23.9–29.8 °C, respectively (Fig. 4). The survey sites were chosen randomly and indecently, irrespective of host plants, thus including both protected cultivation system and open field conditions. The sampled sites had no record of any insecticide application which could affect the whitefly populations. The adult whiteflies were captured from each site with the help of a hand-held aspirator. The captured whiteflies were placed in 1.5 ml Eppendorf tube containing 70% ethanol, labelled with sampling site and date description. The collected insects were stored at − 20 °C until further investigation. Nearly, 100 insects collected from one site were considered as a sample and single individual randomly was chosen for molecular characterization of the species.

**Table 3:** Details of Greenhouse White fly survey along with host plants and geospatial data of collected sites.

S. No	Location	District	State	Host plant	Latitude	Longitude	Altitude (in mtr)	Molecular characterization
1	Hawalbagh	Almora	UK	Salvia	29.634082	79.631259	1229	*T. vaporariorum*
2	Almora	Almora	UK	Marigold	29.589508	79.645099	1626	*T. vaporariorum*
3	Kafligair	Bageshwar	UK	Tomato	29.751903	79.744241	1229	*T. vaporariorum*
4	Doba	Bageshwar	UK	Capsicum	29.805475	79.703495	1606	*T. vaporariorum*
5	Niglat Malla, Kainchi	Nainital	UK	Capsicum	29.401018	79.514373	1758	*T. vaporariorum*
6	Darim	Nainital	UK	Tomato	29.457497	79.635877	1791	*T. vaporariorum*
7	Dwarahat	Almora	UK	Capsicum	29.767179	79.425900	1464	*T. vaporariorum*
8	Govindpur	Almora	UK	Frenchbean	29.68223	79.56524	1323	*T. vaporariorum*
9	Rudradhara	Bageshwar	UK	Ornamental	29.855093	79.567875	1707	*T. vaporariorum*
10	Pantnagar	Udham singh nagar	UK	Tomato	29.027109	79.479966	239	*T. Vaporariorum*
11	Mussoorie	Dehradun	UK	Ornamental	30.480667	78.052262	1684	*T. vaporariorum*
12	Chopta	Rudraprayag	UK	Ornamental	30.489287	79.217040	3455	*T. vaporariorum*
13	Gopeshwar	Chamoli	UK	Capsicum	30.436796	79.320747	1701	*T. vaporariorum*
14	Valley of flowers	Chamoli	UK	Ornamental	30.726323	79.599393	3526	*T. vaporariorum*
15	Kasauli	Solan	HP	Cauliflower	30.898403	76.979865	1630	*T. vaporariorum*
16	Kufri	Shimla	HP	Potato	31.099063	77.264912	2647	*T. vaporariorum*
17	Kasol	Kullu	HP	Potato	32.010927	77.319074	1590	*T. vaporariorum*
18	New Delhi	New Delhi	Delhi	Brinjal	28.641237	77.169133	227	*T. vaporariorum*
19	Lembucherra	West tripura	Tripura	Tomato	23.904222	91.314826	54	*T. vaporariorum*
20	Jal Dhalar Someshwar	Almora	UK	French bean	29.784681	79.602904	1425	*T. vaporariorum*
21	Mehragaon, Bhimtal	Nainital	UK	Ornamental	29.373460	79.557965	1537	*T. vaporariorum*
22	Ranibagh	Nainital	UK	Capsicum	29.286033	79.547461	584	*T. vaporariorum*
23	Nainital	Nainital	UK	Ornamental	29.389418	79.465673	2214	*T. vaporariorum*
24	Pangoot	Nainital	UK	Cauliflower	29.424456	79.427403	1992	*T. vaporariorum*
25	Jageshwar	Almora	UK	Capsicum	29.639179	79.852336	1853	*T. vaporariorum*
26	Daulaghat	Almora	UK	French bean	29.67982	79.56708	1319	*T. vaporariorum*
27	Lakhwada	Dehradun	UK	Snake guard	30.53282	77.96601	1147	*T. vaporariorum*
28	Dhanpau	Dehradun	UK	Tomato	30.53396	77.96121	1231	*T. vaporariorum*
29	Vikas nagar	Dehradun	UK	Brinjal	30.43797	77.73987	448	*T. vaporariorum*
30	Rampur Sheraghat	Pithoragarh	UK	Okra	29.701517	79.904092	740	*T. vaporariorum*
31	Gwaldam	Chamoli	UK	Bottle guard	30.003627	79.566201	1932	*T. vaporariorum*
32	Mukteshwar	Nainital	UK	Garden pea	29.467780	79.632610	1797	*T. vaporariorum*

### DNA extraction, PCR amplification and Sanger sequencing

The standard methodology of CTAB as described by Subbanna et al. was followed for extraction of genomic DNA from an individual whitefly specimen^40^. Each individual whitefly was properly rinsed in sterile distilled water, crushed and homogenized using a hand-held homogenizer (Sigma Aldrich). 120 μl of CTAB solution (20 parts of 1 M Tris–HCl, 8 parts of 0.5 M EDTA, 56 parts of 5 M NaCl and 4 parts of CTAB and the final volume makeup 1000 ml and the pH adjusted to 8. After this 0.4 parts of ß-mercaptaethanol were added along with 1 mg/ml of proteinase K was transferred to the tube and incubated in a water bath at 57 °C for 1–3 h. The contents were vortexed manually after every 20 min for thorough degradation of the tissues. The degraded material was treated twice with phenol–chloroform-isoamyl solution (25:24:1) to extract the genomic DNA and ice-cold isopropyl alcohol was used to precipitate the DNA at − 20 °C for 30 min. The DNA pellet obtained after centrifugation at 10,000 rpm for 7 min was washed with 70% chilled ethyl alcohol to remove the excess salts and was suspended in 20 μl of TE buffer. DNase-free RNase A treatment was followed for 20 min at 37 °C to remove the RNA residues. Electrophoresis with 0.8% agarose gel was carried out to visualize intact genomic DNA. Furthermore, the DNA concentration were quantified in Nanodrop (NanoDrop™ 2000/2000c) subsequently used as template for PCR amplification. The segments of *mtCOI* gene were amplified using pair of universal primers (COX1: LC01490- “5′-GGTCAACAAATCATAAAGATA-3′” and HC02198 “5′-TAAACTTCAGGGTGACCAAAAAA-3′”) with 710 bp product^19,41^. The PCR reaction programme was set using 25 µl reaction mixtures including 12.5 µl of ready to use PCR master mix (Promega M750A), 5.5 µl of nuclease free water, 1 µl each of forward and reverse primer and 5 µl of DNA template. The thermo-cycler programme was run with the following cycles; 94 °C for 5 min as initial denaturation followed by 35 cycles of denaturation at 94 °C for 45 s, annealing at 52 °C for 45 s, extension at 72 °C for 45 s and the final extension step for 10 min at 72 °C. The presence of amplified PCR product was visualized and confirmed in the gel documentation system (Alpha Image Analyzer, Alpha Innotech Corporation) by 1.2% agarose-EtBr 10 mg/ml gel electrophoresis with 2.5 μl PCR product. Gel elution columns (Sigma) were used for purification of the amplified products of the target gene. The purified products were sequenced directly by an automated DNA sequencer (ABI 377) following manufacturers guidelines for the Big Dye terminator kit (Applied Biosystems).

### Similarity search, phylogenetic analysis and population genetics study

The raw reads of *mtCOI* gene sequence were assembled using Clustal Omega (1.2.2) multiple sequence alignment programme^42^. The sequences were exported to NCBI and BLASTn similarity search performed with default parameters. All the available whitefly sequences from databases were retrieved and aligned with ClustalW and bases were edited in BioEdit v7.2.5. Phylogeny was estimated with Mega X^43,44^ following Kimura 2-parameter model and the maximum likelihood approach. To test the phylogenies, a bootstrap replication of 1000 was performed^45^. Genbank accessions *Bemicia tabaci* AY057182.1 was included as outgroups. All the generated sequences from this study have been submitted to the NCBI GenBank with respective accession numbers (Table 1). For each population the sequence polymorphism, singleton variable sites, average nucleotide differences, G+C content, number of haplotypes, haplotype diversity and nucleotide diversity of the *mtCOI* sequences of *T. vaporariorum* were defined using DnaSP v5.10^46^. The popART software was used to analyze the haplotype network of *T. vaporariorum* sequences by constructing a minimum spanning network relationship among the collected samples.

### Evolutionary analysis by Maximum Likelihood method

The evolutionary history was inferred by using the Maximum Likelihood method and Tamura-Nei model^47^. The bootstrap consensus tree inferred from 500 replicates^44^ is taken to represent the evolutionary history of the taxa analyzed. Branches corresponding to partitions reproduced in less than 50% bootstrap replicates are collapsed. The percentage of replicate trees in which the associated taxa clustered together in the bootstrap test (500 replicates) are shown next to the branches. Initial tree(s) for the heuristic search were obtained automatically by applying Neighbor-Join and BioNJ algorithms to a matrix of pairwise distances estimated using the Maximum Composite Likelihood (MCL) approach, and then selecting the topology with superior log likelihood value. This analysis involved 50 nucleotide sequences. Codon positions included were 1st + 2nd + 3rd + Noncoding. There was a total of 883 positions in the final dataset. Evolutionary analyses were conducted in MEGA X^45^.

### Ethics statement

*T. vaporariorum* is not an endangered species that threatens biodiversity. So, no permits were required for collecting it from the field.

## Supplementary Information

Supplementary Information 1.

Supplementary Information 2.

Supplementary Information 3.

Supplementary Information 4.

Supplementary Information 5.

## Data Availability

Data will be made available upon request to corresponding author Dr Ashish Kumar Singh.
