# Supplementary material for: Unravelling geospatial distribution and genetic diversity of greenhouse whitefly, Trialeurodes vaporariorum (Westwood) from Himalayan Region
Source: Sci Rep. 2023 Jul 24;13:11946. doi: 10.1038/s41598-023-37781-y (PMC10366149; doi:10.1038/s41598-023-37781-y)
Supplement: Supplementary file 4 — Supplementary Information 4. [file 41598_2023_37781_MOESM4_ESM.docx]

**Supplementary figure 1. Evolutionary analysis by Maximum Likelihood method**

The output of a molecular evolutionary genetics analysis using MEGA X software version 10.1.7, which is a tool used for analyzing DNA and protein sequence data to infer evolutionary relationships and patterns. The analysis was performed on a nucleotide data set of 56 taxa with 879 sites, and the Tamura-Nei (1993) model was used with first, second, third, and non-coding codon positions selected. The initial tree was automatically made using the NJ/BioNJ method. The analysis statistics show that the sum of branch lengths was 1.658, and the AICc, BIC, and LnL values were 7128.847, 8090.792, and -3450.041, respectively. The Ts/Tv value was 1.062, and the substitution rates between nucleotides were also reported. The phylogenetic tree was constructed using Maximum Likelihood method and included 56 sequences from different isolates of *Trialeurodes vaporariorum* including 32 isolates of current study. The tree topology revealed different clades, where isolates of current study are grouped with each other I most of the cases suggesting their relatedness among each other. The graphical representation of the tree is shown in Figure 1.

**Figure 1**
