# Supplementary material for: Unravelling geospatial distribution and genetic diversity of greenhouse whitefly, Trialeurodes vaporariorum (Westwood) from Himalayan Region
Source: Sci Rep. 2023 Jul 24;13:11946. doi: 10.1038/s41598-023-37781-y (PMC10366149; doi:10.1038/s41598-023-37781-y)

**Supplementary figure 2.** Gel image showing successful amplification of mtCOI gene in collected GWF samples. PCR product was visualized and confirmed in the gel documentation system (Alpha Image Analyzer, Alpha Innotech Corporation) by 1.2% agarose-EtBr 10 mg/ml gel electrophoresis with 2.5 μl PCR product and 1 kb gene ruler (Himedia).


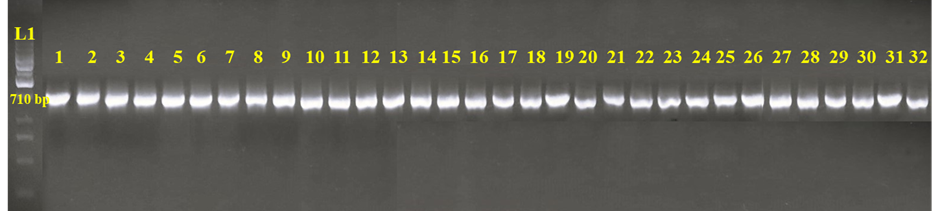

Supplement: Supplementary file 5 — Supplementary Information 5. [file 41598_2023_37781_MOESM5_ESM.docx]
